# k-Shape clustering for extracting macro-patterns in intracranial pressure signals

**DOI:** 10.1186/s12987-022-00311-5

**Published:** 2022-02-05

**Authors:** Isabel Martinez-Tejada, Casper Schwartz Riedel, Marianne Juhler, Morten Andresen, Jens E. Wilhjelm

**Affiliations:** 1 Clinic of Neurosurgery, Copenhagen University Hospital, Rigshospitalet, Copenhagen, Denmark; 2 Department of Health Technology, Technical University of Denmark, Kongens Lyngby, Denmark

**Keywords:** Intracranial pressure, Macro-pattern, k-Shape clustering

## Abstract

**Background:**

Intracranial pressure (ICP) monitoring is a core component of neurosurgical diagnostics. With the introduction of telemetric monitoring devices in the last years, ICP monitoring has become feasible in a broader clinical setting including monitoring during full mobilization and at home, where a greater diversity of ICP waveforms are present. The need for identification of these variations, the so-called macro-patterns lasting seconds to minutes—emerges as a potential tool for better understanding the physiological underpinnings of patient symptoms.

**Methods:**

We introduce a new methodology that serves as a foundation for future automatic macro-pattern identification in the ICP signal to comprehensively understand the appearance and distribution of these macro-patterns in the ICP signal and their clinical significance. Specifically, we describe an algorithm based on k-Shape clustering to build a standard library of such macro-patterns.

**Results:**

In total, seven macro-patterns were extracted from the ICP signals. This macro-pattern library may be used as a basis for the classification of new ICP variation distributions based on clinical disease entities.

**Conclusions:**

We provide the starting point for future researchers to use a computational approach to characterize ICP recordings from a wide cohort of disorders.

## Introduction

Intracranial pressure (ICP) monitoring is a mainstay of neurosurgical diagnostics both for intensive care management in acute neurosurgical conditions [1] and for aiding diagnosis in conditions outside the intensive care unit (ICU) for milder degrees of disease such as hydrocephalus, normal pressure hydrocephalus (NPH), or idiopathic intracranial hypertension (IIH).

In the clinical setting, ICP is often interpreted purely as a number within a certain range. Yet, ICP signals are complex time series with wave patterns that go beyond just a simple number. Analysis of ICP waveforms on either a subsecond beat-to-beat basis or in patterns over longer durations, the so-called macro-patterns, gives further insight into brain function [2]. Machine learning tools have the potential to identify these patterns faster and—more importantly—objectively, helping to characterize their appearance and distribution in a standardized fashion compared to the current primary visual inspection by clinicians. Until now, most studies have employed these techniques to analyze the ICP in acute conditions. Mariak et al. used artificial neural networks (ANN) to extract global properties of the entire ICP time series to assess the severity of the clinical state in intensive care patients [3]. Hornero et al. analyzed the complexity of the ICP signal estimated by approximate entropy (ApEn) to determine the presence of patterns in periods of acute elevations in ICP of pediatric patients in intensive care [4].

In the last decade, new telemetric ICP monitoring devices have become available, allowing easier access to perform ICP recordings that are representative of daily life conditions, compared to previous cable-based solutions [1, 5]. Thus, ICP can now be monitored in patients with milder degrees of disease in disease categories such as hydrocephalus, normal pressure hydrocephalus, or idiopathic intracranial hypertension. The ICP signals recorded with these systems ensure sufficient clinical and technical quality to be analyzed as part of the ICP interpretation procedure carried out by neurosurgeons and other clinicians [6–8], but the increased monitoring period and signal diversity also means that the analysis of ICP data becomes more demanding.

In this study, we explore the use of machine learning tools to extract macro-patterns from the ICP signal in a diverse cohort of patients with different disease entities. We introduce a new methodology based on k-Shape clustering as a basic building block for future day-to-day ICP evaluation and update of models on stored patient data. Given that telemetric ICP monitoring has allowed us to evaluate the patient’s ICP out of hospital borders, our main context for considering new macro-patterns moves away from ICP monitoring exclusively in the neurointensive care setting, where ICP variations are more accentuated. Specifically, our approach aims to permit a more adequate description of the longer timescale ICP variations seen in the broader clinical setting nowadays including disease types like NPH or IIH. Our approach created a universal library of representative macro-patterns that can later be used to automatically segment each individual ICP signal into shorter sequences based on clinical input. Also, we developed a template matching framework to classify these shorter sequences—which we will refer to as ICP subsequences—into what we estimate to be clinically significant macro-patterns. Finally, we propose a possible visualization strategy to display the pattern-annotated ICP signal in a fashion that is clinically useful.

## Methods

**Fig. 1:**
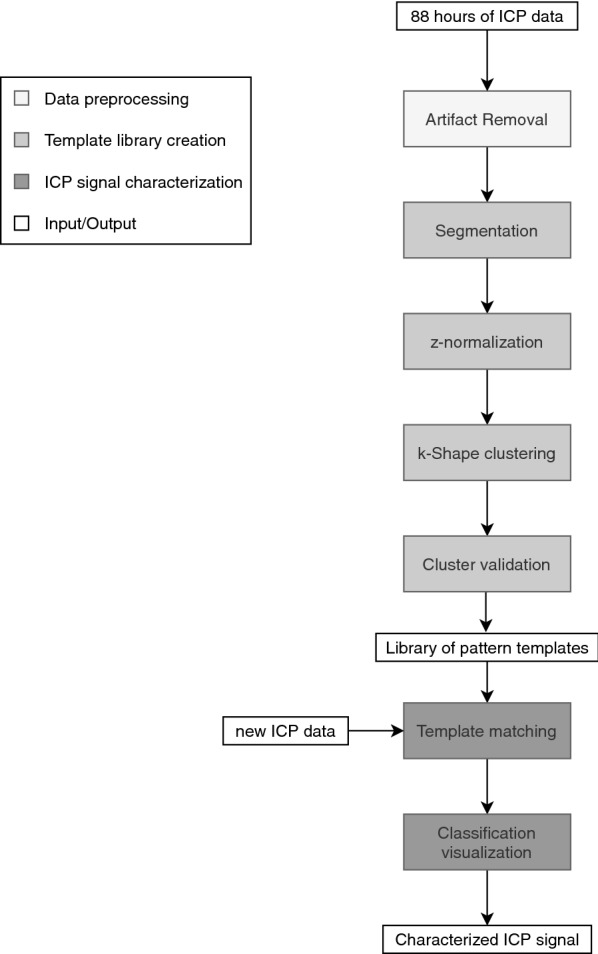
Workflow of methodology developed in this paper for 88 h of ICP data, and an additional data set of 55 h purely for investigating how new incoming ICP data in the future can be labelled

Our goal was to create a scalable library of a few macro-pattern templates to use for ICP subsequence classification. We used k-Shape clustering as a method to efficiently group together subsequences characterized by their shape similarity despite differences in amplitude, duration and alignment. We first describe our data selection and processing approach for artifact removal. Next, we discuss our k-Shape based clustering approach to construct the templates. Finally, we show how the stored library can be used to characterize new incoming ICP signals by reproducible macro-patterns. The components of the entire approach are illustrated in Fig. 1.

### Data selection

We used a collection of eight randomly selected anonymized overnight monitoring sessions that belong to different subjects from our database in the Department of Neurosurgery, Rigshospitalet, Denmark. A commercially available cable ICP probe (Neurovent-P; Raumedic AG, Germany) was used for these measurements. The length of the sessions spanned from nine to 22 h, summing up to a total of 88 h. The sampling frequency of the recordings was 100 Hz. The dataset was made up of five monitoring sessions (five patients) for a total of 88 h, and an additional set of three monitoring sessions (three patients) for a total of 55 h. By adding the latter dataset, template matching results can provide an indication of whether the algorithm is general enough to cover subjects with different disease entities.

### Data preprocessing

The ICP signal recorded is often contaminated by very high and sharp spikes, with unphysiologically high values. These artifacts mask the characteristic appearance of the signal, rendering accurate pattern recognition impossible. We used an Empirical Mode Decomposition (EMD) based method for spike removal [9].

EMD decomposes the signal into a set of intrinsic mode functions (IMFs, i.e., IMF$_1$, IMF$_n$, ..., IMF$_N$). The first function of this set corresponds to fast oscillations, while the last one corresponds to the slowest ones. Therefore, the higher the IMF order, the lower will be its frequency content.The first IMFs, containing high-frequency oscillations, indicate the presence of artifacts. Because the spikes have band-limited waveforms, their dominant oscillations are found in a subset of consecutive IMFs. In our case, the location of unphysiologically high and rapid spikes aligned with the location of spike events in IMF$_{1}$ to IMF$_{4}$, so summing these four IMFs enhances spike episodes. The summation result reveals the peaks with dominant amplitude at the temporal location of the spike, and attenuates the effect of non-spike events. The term $g_r$ will be used to refer to the partially reconstructed signal calculated as the sum of the first to fourth IMFs.

To identify the peak events in $g_r$, an adaptive thresholding approach was implemented. ICP values outside the bounded region between [$-\eta _s$, $\eta _s$] were identified as spikes. The threshold was calculated as $\eta _s= \sigma \sqrt{2\cdot \log (L)}$, where $\sigma$ and *L* are the standard deviation (noise level) and number of samples of $g_r$, respectively. It is a universal threshold first proposed by Donoho and Johnstone [10] for determining a value above background noise. Identified spikes were then imputed with a moving average calculated over a sliding window of 10s.

### Template library creation

We implemented the algorithm in MATLAB (R2020b; The MathWorks, Inc., Natick, MA.) using the platform: Intel®with core i7 processor and clock speed 2.6 GHz and 16 GB RAM.

#### Segmentation

**Fig. 2:**
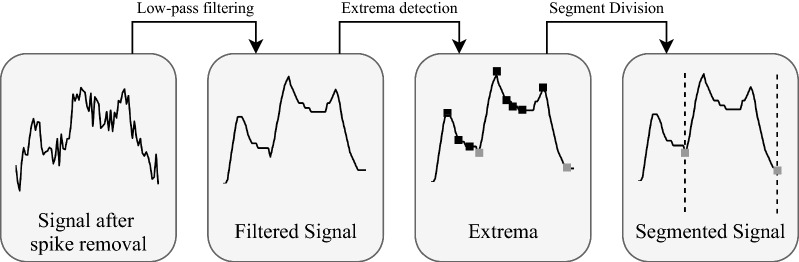
Workflow of the steps involved in the generation of the ICP subsequences, describing the segmentation step in Fig. 1

Time series segmentation plays an important role in data mining and refers to the tool for decomposing the signal into a discrete number of contiguous subsequences. The proposed algorithm for segmentation of the ICP signal can be broken down into four sequential steps, as seen in Fig. 2. The following section will cover the details regarding each of the steps.

ICP segmentation was applied to divide the signal into subsequences of duration varying from seconds to minutes. This poses the challenge of deciding the time location at which to anchor both the start and end points of each subsequence. To address this problem, we first smoothed the signal via a linear phase finite impulse response (FIR) lowpass filter. The filtered signal will only be used in the segmentation step. The cut-off frequency ($F_{pass}$) was set to 0.05–0.1 Hz, depending on the degree of smoothing desired for the removal of cardiac and respiratory contributions in each subject. Other filter parameters were $F_{stop}$ = 0.02–0.05 Hz, $A_{pass}$ = 0.001 dB, $A_{stop}$ = 60 dB, and minimum order.

From the smoothed ICP signal, the major extrema were extracted (maxima and minima). Only the minima were used as the start and endpoints for each of the ICP subsequences. Because some minima were located very close—both time and amplitude wise—to a neighboring maximum, we implemented the following rule to identify suitable minima for the segmentation. If we suppose that the discrete ICP signal at this stage can be written as $g_n$, $n=1,2,...,N$ and indexed in time order $t_n$, $t=1,2,...,N$, we removed a minima $g_i$ from being a candidate as a boundary point if:

1. the time difference between the minimum $g_i$ and its neighboring maximum $g_j$ was smaller than a predefined value $\eta _{dur}$, between 0.5 and 2 min, i.e. $|t_j-t_i|<\eta _{dur}$, or
2. the magnitude difference between a minimum $g_i$ and its neighboring maximum $g_j$ was smaller than a predefined value $\eta _{mag}$, between 0.5 and 1.5, i.e. $|g_j-g_i|<\eta _{mag}$.

We can then define the segmented window (i.e., ICP subsequence) as *g*[*i*, *j*] with *i* and *j* corresponding to the discrete indices of the selected boundary points. An example of these steps is shown in Fig. 5A–C.

#### Z-normalization

Z-normalization of the derived subsequences was required before clustering. As many recent studies [11, 12] suggest, this procedure is necessary for data mining algorithms to deal with scale and translation invariance to prioritize shape features over amplitude ones. By z-normalizing each subsequence we ensured that they were linearly transformed to have zero mean and standard deviation close to one:

1 $$\begin{aligned} z(g[i,j])= \frac{g[i,j]-\mu _{g[i,j]}}{\sigma _{g[i,j]}} \end{aligned}$$

where $\mu _{g[i,j]}$ and $\sigma _{g[i,j]}$ refer to the mean and standard deviation of the ICP subsequence *g*[*i*, *j*], respectively. For the sake of simplicity, we will refer to each z-normalized ICP subsequence *z*(*g*[*i*, *j*]) as $z_{icp}$ in the rest of the paper.

#### k-Shape clustering

k-Shape was used to divide our extracted ICP subsequences into a number of characteristic-preserving groups, the so-called clusters, such that sequences in the same group were similar in shape. Each cluster is represented by a central vector, the centroid, which is not necessarily part of the original dataset [13]. Each centroid in k-Shape is determined as a sequence that minimizes the sum of squared distances to the rest of the z-normalized ICP subsequences. This novel centroid-based clustering algorithm is fundamentally a variant of k-means with a distance measure derived from the cross-correlation coefficient [14]. As a result, one template is built for each centroid and subsequently stored together with a class label.

Through an iterative procedure, k-Shape:

1. assigned each z-normalized ICP subsequence to the centroid with the maximum shape similarity in the assignment step, and
2. updated the centroids based on the new members of each cluster, in the refinement step.

The previous two steps of the algorithm were repeated either until there was no change in cluster configuration or until the maximum number of 100 iterations was reached [14].

Shape similarity was defined by the so-called Shape-Based Distance (SBD):

2 $$\begin{aligned} SBD(\overrightarrow{x},\overrightarrow{\text{c}_\text{k}}) = 1-max_w \left( \frac{CC_w(\overrightarrow{x},\overrightarrow{\text{c}_\text{k}})}{\sqrt{R_0(\overrightarrow{x},\overrightarrow{\text{x}})\cdot R_0(\overrightarrow{c_k},\overrightarrow{\text{c}_\text{k}})}} \right) \end{aligned}$$

where *w* is the position at which the cross-correlation $CC_w(\overrightarrow{x},\overrightarrow{\text{c}_\text{k}})$ between the z-normalized ICP subsequence ($\overrightarrow{x}=z_{icp}$) and the centroid vector of each cluster ($\overrightarrow{\text{c}_\text{k}}$) was maximized; and $R_0$ the geometric mean of autocorrelation of each individual sequence $\overrightarrow{x}$ or $\overrightarrow{c_k}$ [14]. Cross-correlation measures the degree of similarity between two time series, which in our case are $\overrightarrow{x}$ and $\overrightarrow{\text{c}_\text{k}}$, calculated as a function of the displacement of $\overrightarrow{x}$ over $\overrightarrow{\text{c}_\text{k}}$. Cross-correlation adds shift-invariance to the SBD measure and can be computed on sequences of different lengths.

Determining the optimal number of clusters, *K*, is a fundamental challenge within partitional clustering and unfortunately, there is not an ideal approach to identify *K*. Given that we had a large amount of data to be clustered into a number of clusters, and this number was dependent on medical practical experience, the need for an initial estimate of clusters is clear. We relied on a direct method, the so-called silhouette index, as the metric to evaluate the quality of the clustering structure. This metric evaluates the clustering quality based on the similarity between subsequences within the same cluster and across different clusters [15]:

3 $$\begin{aligned} S(i) = \frac{b(l)-a(i)}{max\{b(l),a(l)\}} \end{aligned}$$

In Eq. 3, *a*(*l*) is the average distance between subsequence *l* and every subsequence within the same cluster and *b*(*l*) is the minimum average distance between subsequence *l* and every subsequence in different clusters [16]. The optimal estimate of *K* was the value that maximized the silhouette metric over a range of possible values for *K*. The window of solutions for which the silhouette index was calculated ranged from 5 to 20.

#### Cluster validation

Visual inspection of the clustering results is crucial for verifying the accuracy of the partitioning. However, a visual approach is subject to the level of expertise and subjectivity of the investigator. Thus, visualization needs to be combined with standardized cluster validation indices (CVI) tailored to quantitatively evaluate clustering results. Quantitative evaluation of extracted clusters is not straightforward if there is a lack of annotated data. Thus, we need to rely on internal indices. Conclusions from previous studies have shown that there is no best single CVI in each context [17, 18]. Therefore, multiple validation indices will be used in the validation process: Silhouette Index, Davies–Bouldin index (DBI), and Calinski–Harabasz index (CHI).

Silhouette index, introduced in the previous section, is a common metric to measure how well an object lies within a cluster and our selected internal clustering validation index. DBI is the ratio between the average distance of all subsequences of each cluster to their respective centroids and the distance of the centroids of the two clusters, i.e., the ratio between within-cluster compactness and between-cluster separation [19, 20]:

4 $$\begin{aligned} DBI = \frac{1}{K} \sum _{a=1}^K max \Big \{ \frac{d_a+d_b}{d(c_a,c_b)} \Big \} \, {a\ne b} \end{aligned}$$

where *K* is the number of clusters, *a*, *b* are cluster labels, $d_a,d_b$ the average distance of all subsequences in clusters *a* and *b* to their respective centroids, and $d(c_a,c_b)$ the distance between centroids. Smaller values indicate better clustering results, as clusters are more separated from each other and less disperse within each cluster. To be in line with the rest of CVIs, we use $1-DBI$ for comparison of clustering results and thus higher values indicate better clustering solutions.

CHI relates the sum between the cluster dispersion calculated as the distance, $S_B$, between each within-cluster subsequence and its centroid, to the inter-cluster dispersion calculated as the distance ($S_W$) between each centroid to the global centroid (${\overline{c}}$) [21]:

5 $$\begin{aligned} CHI = \frac{tr(S_B)}{tr(S_W)}\cdot \frac{n_p-1}{n_p-K} \end{aligned}$$

where $S_B$ and $S_W$ are the between and within cluster scatter matrices, respectively, *tr* the trace defined by the sum of the elements of the main diagonal of the scatter matrices, *K* the number of clusters and $n_p$ the number of clustered subsequences. The higher the index value, the better the performance of the clustering.

### Characterization of ICP signals

#### Shape-based template matching

The primary goal was to learn what the distinctive shapes for differentiating pattern clusters from each other were. Therefore, when an uncharacterized ICP subsequence entered into our system, we were able to automatically determine if it belonged to a template from the library of patterns or not. For labeling ICP subsequences based on the generated templates, new ICP subsequences from the additional dataset were retrieved and z-normalized to address scaling invariance. To deal with the horizontal shifts and stretching of the subsequence on the templates, we rescaled the time dimension. Query subsequences were then compared to each template for the closest match. For this comparison, we computed the SBD so that the shape similarity could be measured.

This template matching approach is done under the assumption that all queries must be classified to a template, even if the closest match shows a high SBD. This is why apart from defining our template library, we also defined a rule to ensure that the correlation to the closest match is meaningful. Although this parameter can be specified by the user, a reasonable rule is: $CC(z_{icp},\overrightarrow{c_k}) > 0.50$.

#### Classification visualization

**Fig. 3:**
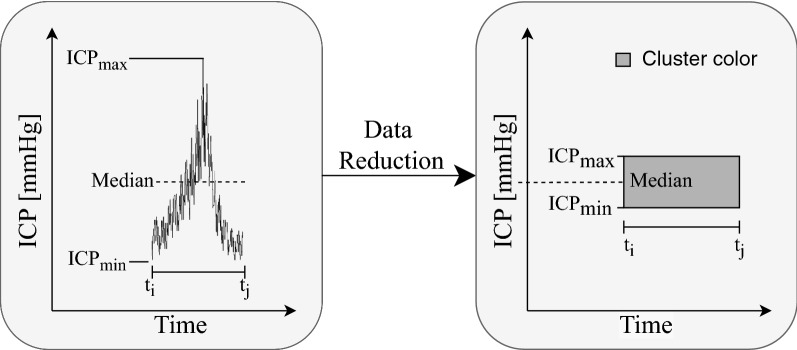
Detailed description of data reduction for each labeled ICP subsequence

The amount of data in each ICP recording is very large. With our current template library, we are able to classify a subset of the ICP signal. Visualizing this information must be presented to a clinical end-user in a fashion that is operationally useful. For this purpose, we represented each ICP subsequence as colored boxes with varying dimensions according to their characteristics (Fig. 3). The height of the box was defined by the difference between the absolute maximum and minimum values of the non-normalized sequence (of the raw unfiltered ICP signal), and the width by the duration of the sequence. The vertical center of the box corresponded to the median ICP value of the non-z-normalized subsequence. Each box was colored after the label their corresponding subsequence had been matched to, being black if the matching correlation coefficient was below 0.50.

## Results

### Data demographics

Eight patients were selected for the study: two male and six female. The pooled median age was 55 years; range: 20–74 years old. Subjects were fetched randomly from a continuously updated clinical ICP database. The clinical conditions were hydrocephalus, aneurysm and craniotomy, but signal analysis was performed on the anonymized recordings without reference to clinical information.

### Data pre-processing

**Fig. 4:**
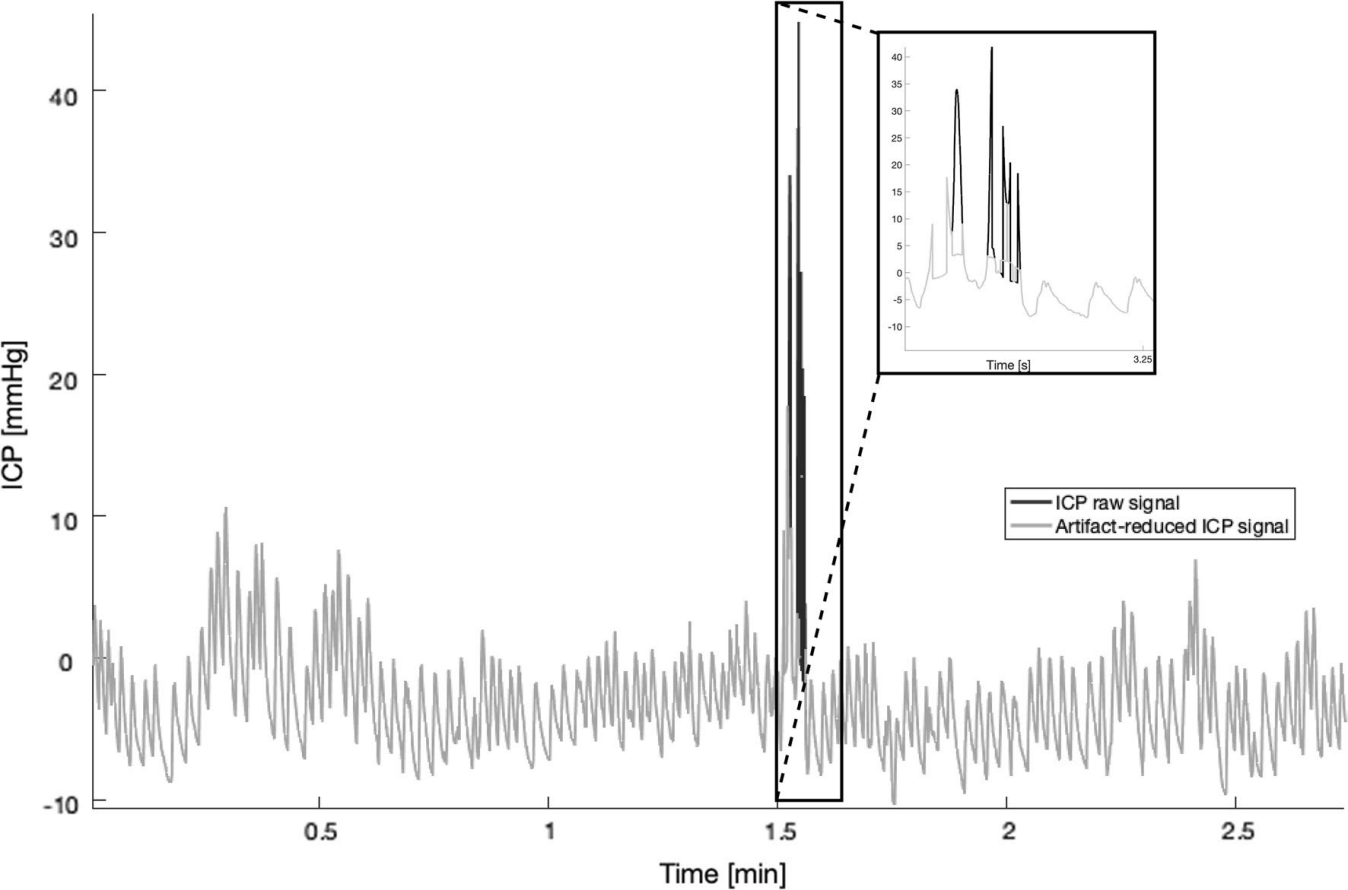
Example of EMD-based filtering of an ICP signal for removal of high spikes

We decomposed the ICP signal via EMD into sixteen IMFs and a residual. Figure 4 shows an example of an ICP signal of one subject after EMD-based filtering, with unphysiologically high and rapid spikes removed.

On average, 18 spikes of less than one second duration are identified in each ICP monitoring. These spikes are found within a range that spans from two to 43 spikes per recording, that account on average for less that 0.000087$\%$ of the total monitoring time. Thus, removing the few samples corresponding to these spikes should not have any major consequences on later processing steps, especially since we will be looking at longer variations of the ICP signal.

### Template library creation

#### Segmentation and normalization

**Fig. 5:**
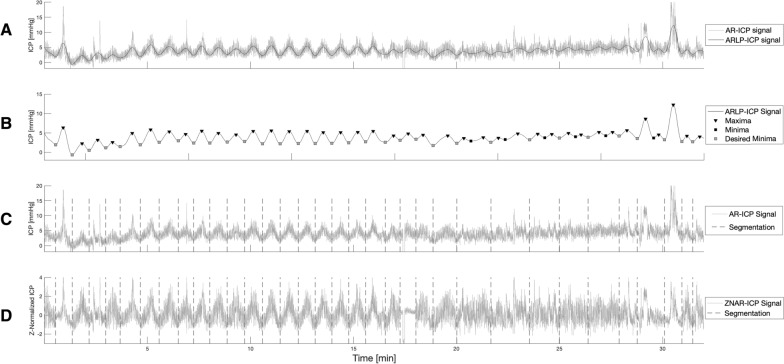
Example of the Artifact removed (AR)-ICP signal segmentation of one subject. **A** After low-pass filtering the AR-ICP signal, with respiratory and pulse contributions to the signal removed to generate the AR-low-pass (ARLP)-ICP signal; **B** After extracted extrema from ARLP-ICP signal; **C** After segmentation using desired minima; and **D** After z-normalization of the segmented AR (ZNAR)-ICP signal

We now show how the ICP signal is segmented and illustrate the segmentation results for the five patients whose recordings made up the main dataset. Figure 5 displays the segmentation process described in Fig. 2. From the figure, we can see that some of the minima extracted, marked as black squares, are not minima that could potentially be considered boundary points. To keep only the minima of our interest, marked as squares, we specified $\eta _{dur}$ and $\eta _{mag}$ for each ICP signal. From the main dataset of 88 h, we were able to generate 5579 ICP subsequences. The last Fig. 5(d) presents how the segmentation results are z-normalized. Z-normalization of a subsequence was done with the mean and standard deviation of that subsequence.

#### k-Shape clustering

**Fig. 6:**
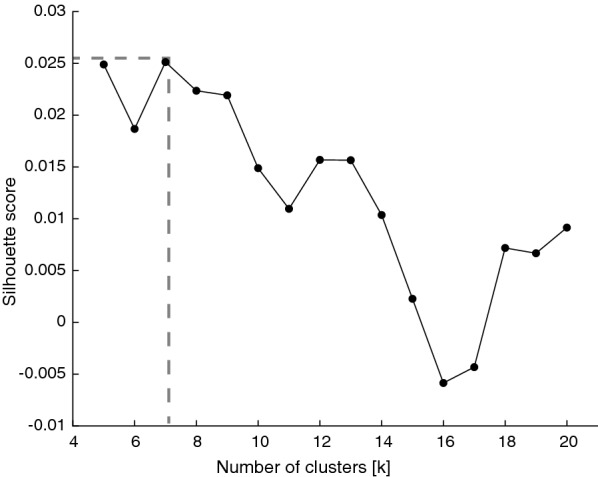
Number of optimal *K* using Silhouette score on the main ICP subsequences

5579 ICP subsequences of varying length generated from the 88 h of the ICP main dataset were clustered with the k-Shape algorithm into seven clusters. The number of optimal clusters to generate the most distinct patterns was calculated using the Silhouette index. We set $K=7$ because it gave us the maximum silhouette value after performing k-Shape clustering for $k=5-20$, as seen in Fig. 6. Going beyond twenty will not contribute to generating distinctive clusters with sufficient information about the ICP data and it will only make our clusters more complicated.

**Fig. 7:**
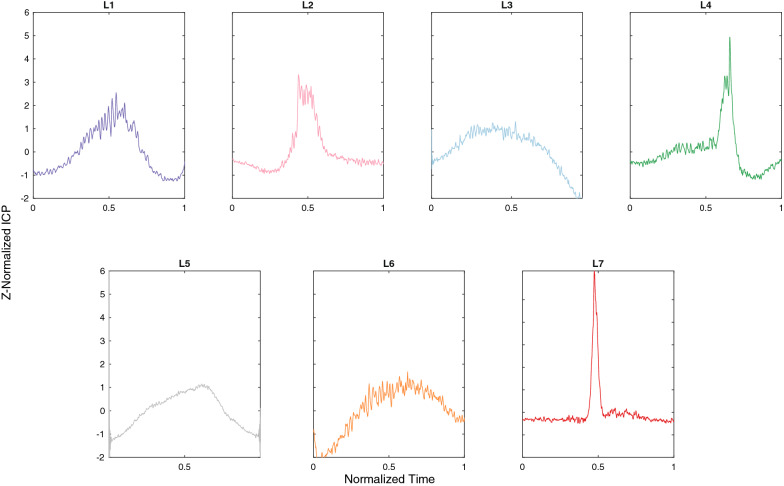
Main extracted reproducible subsequences from the 88 h of ICP recordings (main dataset). These patterns are the foundation for identifying clinically relevant macro-patterns across a wide cohort of patients, moving away from Lundberg’s A and B waves. In contrast to the classical approach, our subsequences could be combined to generate a new macro-pattern. For instance, the ascending *L6* subsequence could be followed by the descending *L3* subsequence, generating a new macro-pattern

To better understand what shapes of centroids were generated, Fig. 7 visualizes the cluster centroids with their corresponding ICP subsequences. This means that the five ICP recordings can be represented by a combination of these seven patterns which can vary in duration and amplitude.

### Cluster validation

**Fig. 8:**
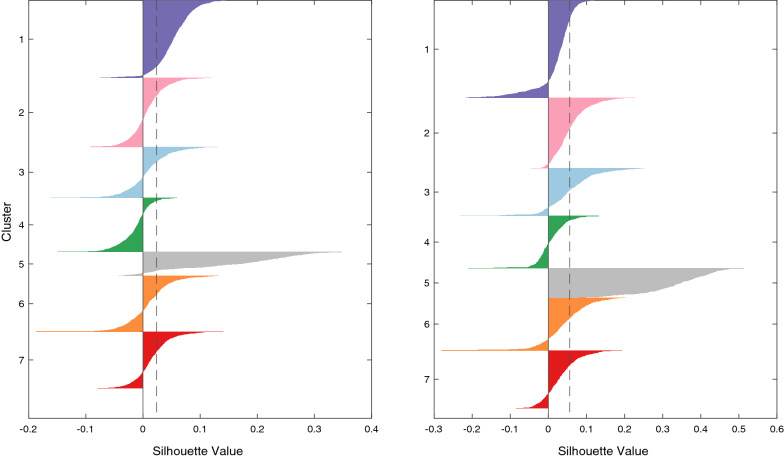
Graphical silhouette values for each clusters when $K=7$ of the main dataset (left), main dataset and correlation rule (right). The dashed vertical line indicates the average silhouette score across all clusters

Silhouette index was used to compare the clustering results of k-Shape applied to the main data with and without the addition of the correlation rule. Figure 8 shows that for Silhouette index, k-Shape together with the correlation rule shows better results over just k-Shape.

The value of the Silhouette index lies within the range − 1 to 1. The closer the index is to 1, the more dense and well-separated from other clusters it is. The addition of the correlation rule increases the silhouette average from 0.024 to 0.056. Another aspect to look for is the thickness (in the vertical axis) of the silhouette plot representing each cluster, being more uniform between clusters when the correlation rule is considered. This idea is reinforced by the results seen in Table 1, with all CVIs increasing when the correlation rule is added.

**Table 1:** Three CVIs for k-Shape clustering without and with correlation rule for the main dataset

CVI	k-Shape	k-Shape with correlation rule
Sil	0.02	0.06
1-DBI	− 9.96	− 8.19
CHI	115.91	123.15

Cluster validation indices highly depend on the complexity of the cluster analysis and on the vague definition of what the nature of the cluster is. Such validation requires a visual approach [22]. We used the additional set to visually confirm that the patterns found in the main set can also be observed in this new data.

### ICP signal characterization

**Fig. 9:**
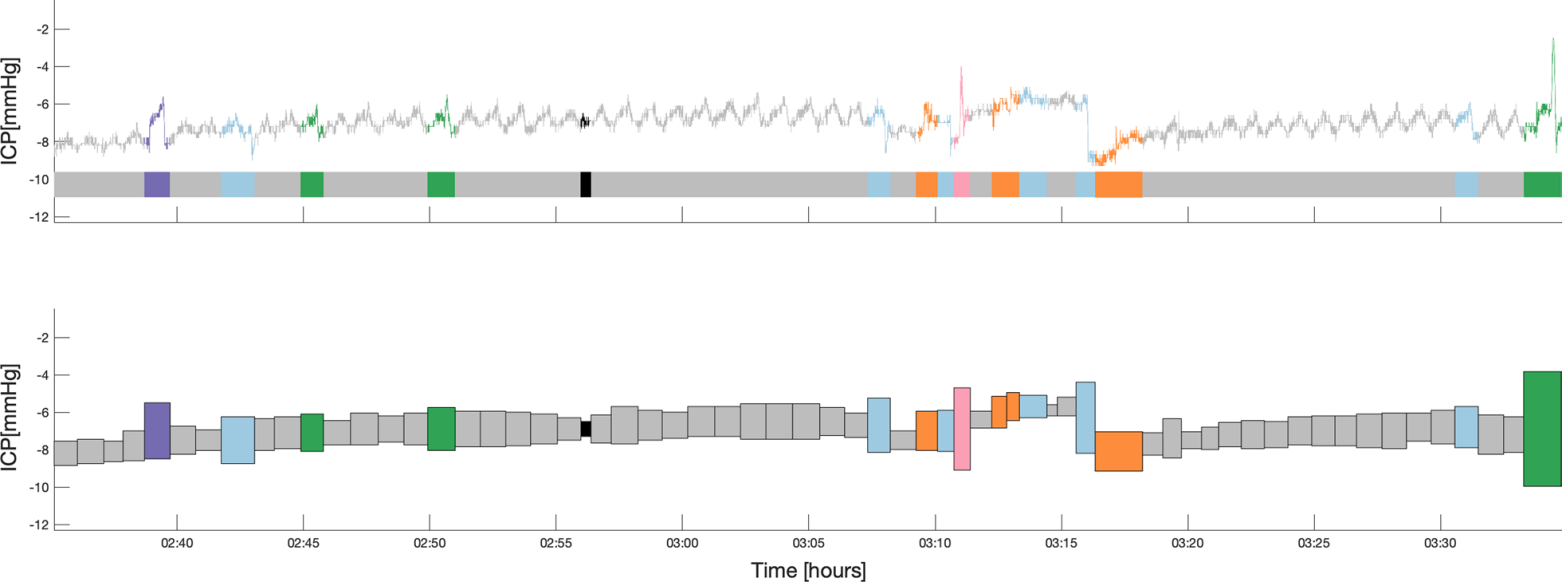
Example of the ICP signal segmentation and classification into labels for one subject visualized in the raw signal (top) and in the data reduced signal representation (bottom)

To allow searching for the minimum distance between each new z-normalized ICP subsequence and each pattern template, we used SBD. Results from the previous section showed the importance of the introduction of a correlation-based rule to ensure that the closest match was significant. We need to bear in mind that if clinicians are our end-users, the ICP template matching output should be clinically intelligible. Figure 9 shows an example of our ICP signal characterization output described in the *Methods* section.

**Fig. 10:**
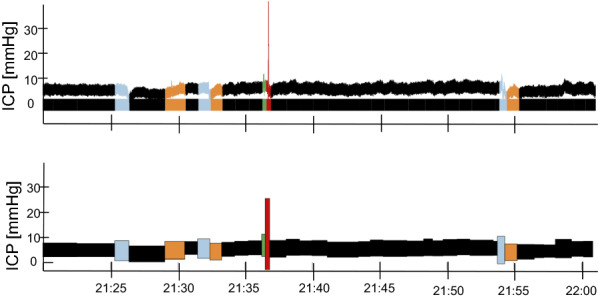
Example of the ICP signal segmentation and classification into labels, including unclassified subsequences, for one subject visualized in the raw signal (top) and in the data reduced signal representation (bottom)

Using the pipeline solution we propose in this paper, we are able to characterize an average of 54$\%$ of the ICP signal. Figure 10 shows an exampled including unclassified ICP subsequences. Further classification details are presented in Table 2.

**Table 2:** Percentage duration of the classified subsequences in the ICP monitoring of each patient. The first five patients correspond to the main set, while the remaining three are part of the additional set

Patient	Monitoring duration (hours)	Duration of classified subsequences (%)
1	8.8	90.4
2	20.1	54.4
3	22.1	52.8
4	17.9	48.6
5	19.3	47.4
6	18.6	50.1
7	18.4	33.0
8	17.7	56.3

**Fig. 11:**
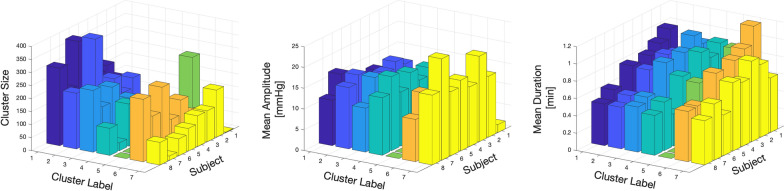
Variation between patients and labels for how often the seven types of patterns occur (left), pattern mean amplitude (middle) and pattern mean duration (right)

Figure 11 provides further visualization of the macro-pattern amplitude and duration in each subject. The presence of specific patterns in unique subjects, in this case *L*5 in Subject 1, could suggest that the occurrence frequency of specific macro-patterns could potentially be used to describe the pathological state of each subject.

## Discussion

Typical A and B waves are described and classified differently by various authors [23] and do no longer adequately address the waveforms encountered in clinical practice today, where patients are investigated also in non-acute scenarios. Therefore, building on top of these classical macro-patterns, a new workflow was developed for the characterization and visualization of long-term ICP variations. Our adaptable pipeline steps includes Empirical Mode Decomposition (EMD) for artifact removal, segmentation into variable-duration subsequences, z-normalization, k-Shape clustering to divide the extracted ICP subsequences into a number of characteristic preserving labels, template-matching to locate the labels in the segmented ICP signal, and finally produces a box-based subsequence labeling display.

A previous study by Paparrizos and Gravano [14] carried out an extensive analysis on the performance of k-Shape against partitional, hierarchical and spectral clustering methods combined with different state-of-the-art distance measures. k-Shape outperformed traditional scalable and non-scalable clustering, such as k-means with Dynamic Time Warping (DTW) as distance metrics or k-medoids, in terms of both accuracy and/or efficiency [14, 24, 25]. Given that we are working with large ICP monitoring sessions, the possibility of scaling while still ensuring high accuracy and efficiency made k-Shape clustering our chosen clustering technique. Unfortunately, k-Shape also presents a limitation: the number of clusters needs to be pre-specified by the user. If the assumed *K* value is above the optimal, the algorithm will generate unnecessary additional groups; if below, we will be under-representing associations between subsequences. There is no perfect method to determine the optimal number, as there is no clear definition of a cluster. We tackled this issue by combining visual inspection with the Silhouette index. Initial visual exploration by clinicians suggested that the search for the optimal *K* should not go beyond $k=20$, as they do not believe in the existence of a number of clinically relevant macro-patterns beyond that value. Thus, the choice of search range was $k = 5-20$, with $K=7$ as the optimal value for our data. We are aware that our methods for selecting *K* are heuristics, and subject to interpretation. A different choice of *K* could yield different results if our study is to be replicated with the same methodology by different research groups.

Besides using CVIs for estimating the quality of the clustering, it is important to visually inspect the results. Clusters *L1*, *L2*, and *L5* could fall into the same category of clinically well-known waveforms since they highly relate to A and B waves. Previous studies classify B waves according to their shape into symmetrical (sinousoidal) and asymmetrical (ramp-like) waveforms [26–29]. Cluster *L1* in our template library resembles the so-called asymmetrical waves, since the duration of the ascending phase is longer than that of the descending. Clusters *L2* and *L5* present more symmetry, with ascending and descending phases of closer duration. They differ in the presence (*L2*) or not (*L5*) of a plateau. For *L2* waveforms, the pressure magnitude will determine its degree of similarity to either A or B waves. Remaining extracted clusters have not been described as such in the literature. Clusters *L6* and *L3* represent ascending and descending segments leading or ending a plateau segment, respectively. This plateau can vary in duration and in some cases contain other clinically relevant macro-patterns. Cluster *L7* is likely to represent subsequences containing artifacts, given the shape of the peak. Finally, cluster *L4* appears as a new non-classified macro-pattern, whose clinical relevance needs to be further analyzed. It must be noted that the templates in our library are normalized in time, meaning that they can be stretched and compressed when matched to incoming ICP subsequences, but constrained by the correlation rule.

With these templates, approximately half of the ICP recording ends up being labeled. This means that for the data considered in this paper, half of it can be represented by just seven shapes (properly scaled horizontally and vertically). This suggests that many ICP signals are often made up of the same patterns repeated again and again. As we have selected to look for one particular macro-pattern type (B wave) to investigate the feasibility of our approach, and the occurrence of macro-pattern types is related to the clinical diagnosis, it is to be expected that the current macro-pattern library does not cover the entire curve length and that the percentage covered can vary between datasets, as we have included these randomly. With this in mind, we have developed the building blocks of a methodology that—with additional retrospective data—could allow identification of previously unencountered macro-patterns in addition to the immediately useful potential of systematic quantitative multidimensional analysis of ICP data. It would be interesting to investigate whether an increased number of templates, K, would increase the fraction of ICP recording being labeled, and especially whether such an increase in the fraction comes at the price of an exponential increase in K. Finally, in this context, one should bear in mind that if K increases to e.g., 100, then the clinical clarity with respect to visual classification might suffer seriously.

The universal scalable library produced so far is the result of combining clinical knowledge of how ICP changes in different clinical conditions, with an engineering approach that moves ICP signal analysis in a more robust quantitative direction with fewer subjective judgments. The results of the present work may be considered benchmarks for the shape clustering method that will be used in our ongoing research. The evident next step is to relate the generated macro-pattern templates with clinical data to ensure that macro-patterns are reproducible and identifiable across a wide cohort of patients with different disease entities. Subsequently, we aim to investigate if it is possible to match disease categories with the occurrence frequency and distribution of the specific macro-patterns. Knowledge of some macro-patterns possibly being more indicative of particular pathological conditions opens opportunities to individualize management and treatment of each patient and obtain a better prediction and understanding of the possible outcome. Furthermore, looking at each label together with additional monitoring of other physiological signals could help to elucidate the origin of each waveform.

The output of our labeling method must be displayed in a way that ensures readability and clarity for clinicians to easily interpret and to integrate it as a new tool in their daily clinical practice. Our visualization strategy is one of the many alternative ways of looking at the raw ICP signal that could be used to accentuate specific features that might not be easily spotted during the visual interpretation of the ICP monitoring. It can be seen as a prototype, among all possibilities of graphical representations, for how the ICP data analysis workflow can be structured. The box approach highlights the presence of the seven identified labels, with many other visualization alternatives yet to be considered, some of them maybe aiming for a report of a certain clinical state. Internal distribution and clinical weighting of the boxes could reflect the pathological state of the patient.

### Limitations

The selection of subjects for the creation of the template library is likely to affect the result, since some patients appear to have more distinguishable macro-patterns than others. Applying this approach to a larger group of subjects is one of the future objectives.

## Conclusions

In this paper, a flexible time series pattern recognition scheme customized to handle ICP time series patterns was introduced. In particular, a clustering algorithm k-Shape clustering was first applied to cluster ICP subsequences to generate a standard scalable library of macro-patterns that can further be used for classification of new incoming ICP signals. We worked with 88 h of ICP recordings and showed the resulting seven clusters that best describe them. Our further research will investigate the clinical use of this technique and look at the practicality of its automatic use to quantitatively interpret ICP data, hoping to reveal a better understanding of the patients underlying physiological status.

## Data Availability

Not applicable.

## Abbreviations

ApEn: Approximate entropy
CHI: Calinski–Harabasz index
CVI: Cluster validation index
DBI: Davies–Bouldin index
EMD: Empirical mode decomposition
FIR: Finite impulse response
ICP: Intracranial pressure
IIH: Idiopathic intracranial hypertension
IMF: Intrinsic mode function
NPH: Normal pressure hydrocephalus
SBD: Shape-based distance
TBI: Traumatic brain injury
